# The role of parental education and occupation on undernutrition among children under five in Bangladesh: A rural-urban comparison

**DOI:** 10.1371/journal.pone.0307257

**Published:** 2024-08-30

**Authors:** Mosammat Zamilun Nahar, Mohammad Salim Zahangir

**Affiliations:** Department of Statistics, University of Chittagong, Chattogram, Bangladesh; University of Memphis, UNITED STATES OF AMERICA

## Abstract

Bangladesh continues to face the persistent issue of child malnutrition. This study aims to investigate the influence of parental characteristics on undernutrition among children under the age of five in both urban and rural areas of Bangladesh. This study utilizes data from the Bangladesh Demographic and Health Survey (BDHS) 2017–18, which includes 7806 children under the age of five and their parents. The effects of parental education and occupation on undernutrition (e.g., stunting, underweight, and wasting) are examined using the binary probit regression technique. Among rural children, 32.6% are stunted, 22.5% are underweight, and 8.1% are wasted. The corresponding figures for urban children are 25.3%, 18.9%, and 8.9%, respectively. In all forms of undernutrition, children living in rural areas face greater challenges than those living in urban areas. The prevalence of stunting and underweight is low among children, irrespective of their rural or urban background, when both parents have the same level of education. The prevalence is also low when fathers are employers or traders and mothers are homemakers, or when highly educated fathers are employers or traders and highly educated mothers are homemakers. In both rural and urban areas, the risk of stunting, underweight and all forms of malnutrition among children decreases as parents’ education levels increase. Children whose fathers work in service or business activities are less likely to experience stunting, being underweight, or wasting compared to children whose fathers work in agriculture or other professions in both areas. In urban areas, children born to mothers with lower levels of education are more vulnerable to wasting than children whose mothers have at least secondary education. To reduce child undernutrition nationwide, it is recommended that both parents have at least a secondary school education and that the father has a stable and sufficient income.

## Introduction

Bangladesh has the highest population density among the world’s largest countries by land area, with 1265 people per square kilometer [1]. The country also faces widespread poverty, with a poverty rate of 21.8% and a low per capita income of 1828 US dollars [2]. The population growth rate in Bangladesh, according to the 2022 census, is 1.08%. The current population growth rate in Bangladesh is concerning, particularly given the country’s high population density and low-income status. To achieve development, it is necessary to reduce this rate even further to ensure a healthy population. Improved health is a key contributor to economic progress, as healthy individuals tend to live longer, be more productive, and save more [3]. Providing adequate nutrition during the earliest stages of life is crucial for promoting physical and mental health and overall well-being.

UNICEF’s 2020 report states that 22% of children under five worldwide suffer from stunting and 6.7% from wasting [4]. The majority of stunted and wasted children live in lower- and middle-income countries. South Asia ranks 3rd for stunting and 1st for wasting in the world [5]. Malnutrition is responsible for the deaths of approximately 3.1 million children under five years old every year worldwide [6]. According to WHO [7], around 45% of children under the age of five are affected by nutrition-related factors. The report also indicates that severely malnourished children face a higher risk of death from common childhood diseases, such as diarrhoea, pneumonia, and malaria. In 2018, two regions, Sub-Saharan Africa and Central and South Asia, accounted for over 80% of the 5.3 million deaths among children under five [7].

Childhood undernutrition has multifaceted and complex causes. For instance, primary factors contributing to undernutrition in children under five include inadequate food intake, limited food access, poor health facilities, lack of safe water and sanitation, and inadequate care for children and mothers [8]. Undernourished children under five not only face a higher risk of mortality, but also suffer severe and adverse consequences in adulthood. According to UNICEF [8], individuals who experience undernutrition during childhood tend to develop low physical and intellectual capabilities, low productivity, and high levels of chronic diseases and disability. This is often observed in societies that lack the economic resources to provide essential therapeutic and rehabilitative services. Undernutrition is worsened by various factors, including poverty, lack of education, food insecurity, inadequate sanitation services, and unhealthy home environments [9–13].

The nutritional status of a child is impacted by their place of residence. Research has shown that children living in rural areas experience higher rates of undernutrition compared to those living in urban areas [10, 14, 15]. This is likely due to the higher poverty rates [16] and lower adult literacy rates [17] found in rural areas. Additionally, rural populations face greater difficulties in accessing education, employment, income opportunities, and communication than their urban counterparts. However, girls from both rural and urban areas who suffer from undernutrition are at an increased risk of becoming undernourished mothers and giving birth to low-birth-weight babies [18].

Several studies have shown a correlation between maternal education and children’s nutrition levels [19–21]. It has been found that girls who receive little or no formal education are more vulnerable to undernutrition. Conversely, educated mothers tend to ensure proper nutrition and medical care for their children. Semba et al. [22] found that parents with higher education are more effective in protecting their children from childhood undernutrition. Smith and Haddad [23] found that increasing maternal education in developing countries can reduce child undernutrition by about 43%. Higher levels of child nutrition are associated with better employment outcomes for fathers [24] and working mothers [11]. Parents with higher education and income tend to show more concern for their children’s well-being [25].

In patriarchal societies like Bangladesh, fathers often take a leading role in family decision-making, while mothers typically manage the household management and childcare. Thus, a child’s health is largely dependent on maternal care, which is closely linked to the mother’s understanding of basic principles of nutrition and healthcare. In addition to mothers, fathers are also responsible for their children’s nutritional status [24, 25]. Therefore, it is crucial to explore the relationship between parental characteristics and their children’s nutritional status.

Several studies on child undernutrition in Bangladesh exist in the literature [21, 26–28]. Some studies assess undernutrition in children under five by considering a limited number of factors, while others explore the relationship between parental education and child undernutrition. Few studies address urban-rural differences in child undernutrition.

This study aims to investigate the relationship between parental characteristics and undernutrition among children under five years of age in both urban and rural areas of Bangladesh. A wide range of variables will be considered as potential confounding factors. Additionally, the study will observe the prevalence and disparities of undernutrition among children under the age of five in both urban and rural areas.

## Methods

### Data sources

This study used a secondary dataset obtained from a nationally representative survey entitled Bangladesh Demographic and Health Survey (BDHS) 2017–18. The survey was conducted through a collaborative effort of National Institute of Population Research and Training (NIPORT) and ICF from October 2017 to March 2018. For the survey, a sampling frame was formed from the list of enumeration areas (EAs) of the 2011 Population and Housing Census of the People’s Republic of Bangladesh, provided by the Bangladesh Bureau of Statistics (BBS) and a two-stage stratified random sampling technique was used. Initially, 675 EAs (250 in urban areas and 425 in rural areas) were selected with the probability proportional to the size of EAs and subsequently a sample of 30 households, on average, were selected per EA. Finally, a total of 20,127 ever-married women of age ranging from 15 to 49 were fruitfully interviewed from the overall selected 20,160 residential households from all (eight) administrative divisions. These women had 8,759 children between the ages of 0 and 59 months (e.g., children under 5) at the time of the interview. Out of those, 7,806 children were included in the study, while the remaining 953 were excluded due to various reasons, including missing data, flagged cases and plausible limitations.

### Study variables

#### Dependent variable

The term malnutrition refers to three broad groups of conditions. One is undernutrition, which includes wasting (low weight-for-height), stunting (low height-for-age) and underweight (low weight-for-age). The others are micronutrient deficiencies or insufficiencies (lack of important vitamins and minerals) and overweight, obesity and diet-related non-communicable diseases such as heart disease, stroke, diabetes, and cancer. Malnutrition refers to undernutrition in this study, wherein wasting, stunting and underweight were used to assess nutritional status. It is important to note that in the 2017–18 BDHS dataset, the weight and height of children under five are assessed to calculate their anthropometric indices, including height-for-age, weight-for-age, and weight-for-height. These indices are expressed in standard deviation units (z-scores) relative to the median of the reference population. NIPORT et al. [29] defines:

- Stunting as height-for-age z-score that is less than minus two standard deviations (-2SD) from the median of the reference population;
- Underweight as weight-for-age z-score that is less than minus two standard deviations (-2SD) from the median of the reference population; and
- Wasting as a weight-for-height z-score that is less than minus two standard deviations (-2SD) from the median of the reference population.

#### Independent variables

In this study, covariates are chosen taking into account both the availability of data and the existing literature on the nutritional status of children under the age of five. These are mother’s age, administrative division, wealth index, parent’s (mother and father) education and occupation, access to mass media, age at first marriage, age at first birth, body mass index, child age, birth order and birth interval. Among these variables, parental education and occupation are considered the main predictors and the remaining covariates are potential confounders. All the covariates and their suitable categories are mentioned in Table 1.

**Table 1:** List of independent variables, their types and suitable categories, and no. of respondents in urban and rural areas corresponding to categories of each variable.

Independent variable	Type of variable	Suitable categories	No. of respondents in	
			Urban	Rural
*Mother’s age (in years)*	Continuous	< 20	296	670
		20–29	1640	3268
		30–49	723	1209
*Division*	Categorical	Barisal	248	571
		Chittagong	434	829
		Dhaka	606	498
		Khulna	305	523
		Mymensingh	237	689
		Rajshahi	239	571
		Rangpur	270	629
		Sylhet	320	837
*Mother’s education*	Discrete	No education	183	375
		Primary	668	1593
		Secondary and above	1808	3179
*Father’s education*	Discrete	No education	343	941
		Primary	770	1864
		Secondary and above	1546	2342
*Mother’s working status*	Categorical	Working	867	2310
		Not working	1792	2837
*Father’s occupation*	Categorical	Service/Business	1674	3999
		Agriculture/Others	985	1148
*Access to mass media*	Combination of three variables: frequencies of listening to the radio, watching TV and reading newspapers at least once in a week.	No access	549	2279
		Have access	2110	2868
*Wealth Index*	A composite measure of a household’s cumulative living standard, which is computed by using principal component analysis.	Poor	519	2797
		Middle	1083	1886
		Rich	1057	464
*Age at first marriage (in years)*	Continuous	≤15	904	2178
		16–19	1304	2496
		20 and above	451	473
*Age at first birth (in years)*	Continuous	≤15	326	810
		16–19	1341	2990
		20 and above	992	1347
*Mother’s BMI* *	Continuous	Thin	322	836
		Normal	1384	3239
		Obese	944	1061
*Child age (in months)*	Continuous	0–11	543	1088
		12–23	532	1058
		24–35	506	1011
		36–47	502	958
		48–59	576	1032
*Birth order*	Discrete	First	1082	1898
		Second	906	1634
		Third and above	671	1615
*Number of living children*	Discrete	1–2	2008	3560
		3–11	651	1587
*Birth interval (in months)* *	Continuous	First birth	1089	1905
		Less than 24	159	359
		24 to 47	450	1023
		48 and above	959	1854
**Total**			2659	5147

*Indicates that some values, such as 20 are missing in maternal BMI and 8 in birth interval.

### Statistical analysis

Both bivariate and multivariate analyses are used as diagnostic tools of data. Bivariate analysis is performed to observe an association between each covariate and nutritional status of children under 5 years of age. Besides, binary probit regression analysis is performed to examine the impact of parental characteristics by confounding a long list of covariates on ‘nutritional status’—a dichotomous variable (e.g., non-stunting or stunting; healthy weight or underweight; and non-wasting or wasting). Indeed, the probit model as a discrete choice model is appropriate for a dichotomous variable [30]. Suppose a response variable Y takes two possible outcomes: presence or absence of a certain condition. In this study, Y is formulated as

$$Y=\left\{ \begin{array}{l} 1;\;if\;a\;child\;is\;stunting\;or\;underweight\;or\;wasting\\ 0;\;if\;a\;child\;is\;non‐stunting\;or\;normal\;or\;non‐wasting \end{array}\right.$$

and X = X_1_,X_2_,….,X_*k*_ as a vector of covariates. Then Y is modelled as

$$\mathrm{Pr}\left(Y=1|X\right)=\phi (X^{/}\beta )$$

Where β = β_1_,β_2_,…,β_k_ is a vector of parameters or probit coefficients to be estimated by maximum likelihood. In the model, ϕ is the cumulative distribution function of the standard normal distribution and X^/^β is the probit score or index. A positive value of β can be interpreted as an increase in the probit score by b standard deviations with a one-unit increase in the predictor. Alternatively, it can be interpreted as the more likely to be a child malnourished with the predictor and vice-versa for a negative value.

### Ethical approval

Ethical approval is not required to use the BDHS data as the survey was approved by the local ethics committee in Bangladesh and the ICF Macro ethics committee in Calverton, New York, USA. Details of the ethical approval can be found in the BDHS 2017–18 summary report [29].

## Results

### Bivariate analysis (*prevalence of child undernutrition by background characteristics)*

Table 2 represents the prevalence of undernourishment among children under the age of 5, categorized by child and parental characteristics, in both urban and rural areas of Bangladesh. Among rural children, 32.6% are stunted, 22.5% are underweight, and 8.9% are wasted. The corresponding figures for urban children are 25.3%, 18.9%, and 8.9%, respectively. Moreover, children residing in urban areas experience lower rates of undernutrition compared to those in rural areas. In Table 2, the prevalence of any form of undernutrition is 33.4% in urban areas and 40.2% in rural areas. Similarly, in Table 3, the prevalence of all forms of undernutrition is 2.3% in urban areas and 2.9% in rural areas.

**Table 2:** Percentage of undernutrition in children under five in urban and rural areas by selected characteristics of children and their parents, BDHS 2017–18.

Covariates	Stunting		Underweight		Wasting		Any form of undernutrition	
	Urban	Rural	Urban	Rural	Urban	Rural	Urban	Rural
*Mother’s age*	(0.001)	(0.122)	(0.236)	(0.030)	(0.933)	(0.411)	(0.012)	(0.167)
< 20	34.9	31.9	22.8	19.8	8.3	9.1	41.9	38.5
20–29	24.5	31.9	18.2	22.2	9.0	8.2	32.1	39.7
30–49	22.9	34.9	18.5	24.7	8.8	7.5	33.0	42.3
*Division*	(0.018)	(0.000)	(0.248)	(0.000)	(0.349)	(0.131)	(0.046)	(0.000)
Barisal	22.2	34.9	16.7	22.7	6.9	9.3	29.2	42.5
Chittagong	29.0	33.6	21.2	20.4	7.1	7.8	36.7	40.3
Dhaka	22.7	27.4	17.6	18.7	10.5	7.4	32.0	34.9
Khulna	23.2	26.3	13.1	20.5	5.9	8.5	26.8	34.2
Mymensingh	30.6	35.8	23.4	26.3	10.7	8.9	41.4	42.9
Rajshahi	25.0	32.4	20.6	23.2	9.4	7.4	31.9	41.3
Rangpur	23.4	31.4	18.7	20.4	8.9	6.8	32.5	37.7
Sylhet	38.0	43.2	22.8	33.8	6.5	11.2	43.5	53.4
*Mother’s education*	(0.000)	(0.000)	(0.000)	(0.000)	(0.004)	(0.079)	(0.000)	(0.000)
No education	35.1	45.7	32.3	36.8	14.2	10.9	48.4	54.2
Primary	34.8	40.0	25.6	26.3	10.9	8.3	43.9	46.7
Secondary and above	20.6	27.6	14.8	19.1	7.5	7.7	27.8	35.5
*Father’s education*	(0.000)	(0.000)	(0.000)	(0.000)	(0.120)	(0.393)	(0.000)	(0.000)
No education	37.4	43.8	28.3	30.1	10.2	9.2	46.0	51.0
Primary	33.2	36.4	23.6	24.3	10.5	7.8	40.9	43.7
Secondary and above	18.6	25.4	14.4	18.2	7.8	8.0	26.9	33.4
*Mother’s working status*	(0.001)	(0.001)	(0.000)	(0.072)	(0.136)	(0.750)	(0.000)	(0.026)
Working	29.9	35.0	23.3	23.6	10.3	8.0	38.9	41.8
Not working	23.3	30.7	16.8	21.6	8.2	8.3	31.0	38.9
*Father’s occupation*	(0.000)	(0.000)	(0.001)	(0.000)	(0.025)	(0.000)	(0.000)	(0.000)
Service/Business	18.5	26.1	14.9	17.7	7.0	5.5	26.3	31.3
Agriculture/Others	29.1	34.5	21.1	23.9	9.9	8.9	37.5	42.7
*Access to mass media*	(0.000)	(0.000)	(0.005)	(0.000)	(0.999)	(0.221)	(0.000)	(0.000)
No access	35.3	37.7	23.9	26.1	8.9	8.7	42.8	45.5
Have access	23.0	29.0	17.7	19.9	8.9	7.8	31.3	36.5
*Wealth index*	(0.000)	(0.000)	(0.000)	(0.000)	(0.005)	(0.179)	(0.000)	(0.000)
Poor	36.7	39.0	28.2	26.6	10.3	8.8	44.8	46.4
Middle	29.4	28.1	22.2	19.6	10.8	7.4	39.3	35.3
Rich	17.4	16.0	12.4	11.5	6.6	8.0	23.9	25.8
*Age at first marriage*	(0.000)	(0.011)	(0.000)	(0.521)	(0.245)	(0.264)	(0.000)	(0.007)
≤15	29.6	33.9	23.2	23.2	10.3	8.6	38.2	41.3
16–19	25.9	32.4	17.8	22.0	8.2	8.1	33.4	40.3
20 and above	14.0	27.0	12.8	21.8	7.7	6.4	23.5	33.7
*Age at first birth*	(0.000)	(0.003)	(0.000)	(0.007)	(0.052)	(0.146)	(0.000)	(0.001)
≤15	30.0	35.4	25.9	25.8	9.6	8.4	40.4	42.3
16–19	28.8	33.3	20.1	22.5	10.2	8.6	36.8	41.4
20 and above	19.0	29.2	14.6	20.3	6.9	6.9	26.5	36.1
*Mother’s BMI*	(0.000)	(0.000)	(0.000)	(0.000)	(0.000)	(0.000)	(0.000)	(0.000)
Thin	41.0	40.5	33.0	30.8	15.4	13.2	52.1	50.2
Normal	26.5	32.8	20.2	22.3	9.5	7.5	35.4	40.4
Obese	19.0	25.9	13.0	17.0	6.3	6.7	25.5	32.2
*Child age (in month)*	(0.000)	(0.000)	(0.005)	(0.000)	(0.954)	(0.468)	(0.001)	(0.000)
0–11	17.2	20.4	13.3	14.9	9.7	7.5	26.9	29.2
12–23	28.0	35.4	17.6	18.5	8.9	7.8	34.7	41.1
24–35	31.2	41.3	20.8	24.8	8.5	7.5	38.0	47.6
36–47	29.1	35.3	23.2	26.9	8.2	9.0	38.5	43.8
48–59	21.4	31.2	19.6	28.2	9.1	8.9	29.4	39.8
*Birth order*	(0.015)	(0.000)	(0.005)	(0.001)	(0.350)	(0.876)	(0.006)	(0.000)
First	25.3	30.1	17.3	20.8	7.9	8.4	32.4	37.7
Second	22.1	30.5	17.1	21.6	9.1	8.0	30.7	38.7
Third and above	29.5	37.6	23.8	25.4	10.2	8.0	39.1	44.7
*Number of living children*	(0.043)	(0.000)	(0.002)	(0.000)	(0.317)	(0.772)	(0.011)	(0.000)
1–2	24.2	30.4	17.3	20.9	8.5	8.1	31.9	38.0
3–11	28.7	37.7	23.6	26.2	10.0	8.3	38.2	45.2
*Birth interval*	(0.002)	(0.000)	(0.001)	(0.000)	(0.386)	(0.587)	(0.008)	(0.000)
First birth	25.3	30.2	17.4	20.9	8.0	8.3	32.4	37.6
Less than 24	34.5	39.2	31.0	23.7	11.5	6.5	43.4	44.9
24 to 47	30.1	38.2	22.1	28.2	10.6	8.6	38.6	45.1
48 and above	21.5	30.8	17.1	20.9	8.9	8.0	30.8	39.3
**Total**	25.3	**32.6**	18.9	**22.5**	**8.9**	**8.1**	33.4	40.2

***Note*:** p-values are in parentheses.

**Table 3:** Percentage of undernutrition in children under five in rural and urban areas based on homogamy/heterogamy in parental education, BDHS 2017–18.

Difference in education levels	Stunting	Underweight	Wasting	Any form of undernutrition	All forms of undernutrition
	*In urban areas*				
Father < Mother	32.3	21.8	8.4	39.4	2.8
Father = Mother	22.5	17.0	8.5	30.2	2.2
Father > Mother	27.7	24.1	12.1	41.1	2.2
**Total**	25.3	18.8	8.9	33.4	2.3
	*In rural areas*				
Father < Mother	37.1	23.7	7.7	44.1	2.9
Father = Mother	29.0	21.0	8.1	37.1	2.6
Father > Mother	38.7	27.5	9.8	45.9	4.8
**Total**	32.6	22.5	8.2	40.2	2.9

Table 2 shows the factors that are significantly associated (p-value ≤ 0.05) with stunting. These factors include administrative division, mother’s education, father’s education, mother’s working status, father’s occupation, access to mass media, wealth index, mother’s age at first marriage, age at first birth, mother’s BMI, child age, birth order, number of living children, and birth interval in both urban and rural areas, as well as mother’s age in urban areas. The factors linked to underweight and any form of undernutrition are similar to those associated with stunting. Only mother’s education, father’s occupation, and mother’s BMI show significance (p-value < 0.10) in both rural and urban areas regarding wasting. In urban areas, wealth index and mother’s age at first birth also emerge as significant factors.

The results for the wealth index are particularly interesting when compared with those for all other factors. The prevalence of stunting, underweight and wasting is higher among urban children in different wealth index categories than rural children. For example, within middle-class and rich families, children residing in urban areas exhibit higher rates of stunting (about 1.3% higher among middle-class families and 1.4% higher among rich families), underweight (2.6% higher among middle-class families and 0.9% higher among rich families) and wasting (1.5% higher among poor families and 3.4% higher among middle-class families) than their rural counterparts. In case of any form of undernutrition, middle-class urban children are the most affected (by 4.0% among the middle-class).

### Multivariate analysis

#### Prevalence of child undernutrition by homogamy/heterogamy in parental education

The effects of interaction between parental (mother and father) education and occupation on childhood undernutrition are shown in Tables 3–6. In Table 3, the prevalence of stunting is lowest in both urban and rural children when parents have the same level of education, with rates of 22.5% in urban areas and 29.0% in rural areas. The second lowest prevalence is observed in urban areas when fathers have a higher level of education than mothers (27.7%) and in rural areas when mothers have a higher level of education than fathers (37.1%). Children of parents with the same level of education have the lowest rates of underweight and any form of undernutrition in both urban and rural areas. Conversely, in urban areas, children of mothers with lower levels of education than their fathers have the highest rates, while in rural areas, children of fathers with lower levels of education than their mothers have the highest rates. Children, whether they live in rural or urban areas, are less affected by all forms of undernutrition when their parents have the same level of education (2.6% vs. 2.2%).

**Table 4:** Percentage of undernutrition in children under five in urban and rural areas by parental occupation, BDHS 2017–18.

Father’s occupation	Mother’s working Status	Stunted	Underweight	Wasting	Any form of undernutrition	All forms of undernutrition
		*In urban areas*				
Service/Business	Working	21.9	15.8	6.0	30.6	0.5
	Not working	17.4	14.7	7.5	24.9	1.6
Agriculture/ Others	Working	32.8	26.3	12.0	42.4	3.9
	Not working	27.1	18.3	8.8	35.0	2.2
		*In rural areas*				
Service/Business	Working	26.0	18.2	6.1	30.9	2.9
	Not working	26.0	17.3	5.0	31.7	1.4
Agriculture/ Others	Working	37.3	25.0	8.5	44.6	3.4
	Not working	32.2	23.0	9.2	41.2	3.0

**Table 5:** Percentage of undernutrition in children under five in urban and rural areas by maternal education and occupation, BDHS 2017–18.

Maternal education	Mother’s working Status	Stunted	Underweight	Wasting	Any form of undernutrition	All forms of undernutrition
		*In urban areas*				
No education	Working	43.1	41.5	20.0	55.4	7.7
	Not working	30.0	25.6	10.0	43.3	1.1
Primary	Working	36.0	27.5	13.0	47.0	3.5
	Not working	34.0	24.4	9.6	42.0	3.1
Secondary and above	Working	24.1	18.0	7.1	31.7	1.9
	Not working	19.2	13.6	7.7	26.3	1.8
		*In rural areas*				
No education	Working	47.5	37.2	8.3	53.9	4.1
	Not working	43.5	36.4	14.1	54.9	6.0
Primary	Working	41.4	28.2	8.8	48.3	4.2
	Not working	38.8	24.6	7.9	45.3	3.3
Secondary and above	Working	29.6	19.1	7.5	36.4	2.7
	Not working	26.2	19.1	7.9	34.9	2.0

**Table 6:** Percentage of undernutrition in children under five in urban and rural areas by paternal education and occupation, BDHS 2017–18.

Father’s education	Father’s occupation	Stunted	Underweight	Wasting	Any form of undernutrition	All forms of undernutrition
		*In urban areas*				
No education	Service/ Business	32.8	27.9	11.5	42.6	3.3
	Agriculture/ Others	38.7	28.4	10.3	47.1	2.9
Primary	Service/ Business	30.9	22.8	9.7	38.2	4.1
	Agriculture/ Others	33.7	23.8	10.7	41.5	4.0
Secondary and above	Service/ Business	14.2	11.7	6.1	21.7	0.7
	Agriculture/ Others	22.5	16.6	9.3	31.3	2.1
		*In rural areas*				
No education	Service/ Business	33.3	28.6	8.7	39.7	5.6
	Agriculture /Others	45.2	30.3	9.3	52.7	4.0
Primary	Service/ Business	36.9	21.7	5.7	40.7	2.7
	Agriculture /Others	36.4	24.8	8.2	44.3	2.7
Secondary and above	Service/ Business	20.6	14.5	4.8	26.2	1.2
	Agriculture/ Others	27.5	19.9	9.4	36.5	3.3

#### Prevalence of child undernutrition by interaction between fathers and mothers occupation

In Table 4, the prevalence of stunting and underweight is significantly lower in rural and urban children whose fathers work as service owners or businessmen and mothers are homemakers, with rates of 17.4% compared to 26.0% for stunting and 14.7% compared to 17.3% for underweight. Rural children whose parents have the same standards have lower rates of stunting (5.0%) and all forms of undernutrition (1.4%), while urban children have lower rates of all forms of undernutrition (24.9%). In addition, children in rural areas with fathers engaged in business or service sectors and working mothers have a lower incidence of any form of undernutrition (30.9%), while children in urban areas also have a lower risk of undernutrition in all its forms (0.5%). Children living in urban areas whose fathers are farmers or other professionals and mothers are manual laborers face the most severe nutritional challenges according to all nutrition assessments. Similar circumstances can be found in all nutritional evaluations, except for wasting, among rural children whose parents have the same standards.

#### Prevalence of child undernutrition by interaction between maternal education and occupation

Table 5 shows that in urban areas, children whose mothers have no formal education and are working have a higher risk of being undernourished, based on all nutritional assessments. In contrast, children whose mothers have secondary or higher education and are not working have lower rates of stunting (19.2%), underweight (13.6%), and any form of undernutrition (26.3%). A similar pattern is evident among rural children suffering from stunting and underweight. Amongst rural children, those with illiterate or working mothers have the highest prevalence of wasting (14.1%), undernutrition in any form (54.9%), and undernutrition in all forms (6.0%). The incidence of wasting is negligible among rural and urban children when their mothers have at least secondary education and are actively engaged in the labor force (7.5% vs. 7.1%). The prevalence of all forms of undernutrition is exceptionally low, only 1.1%, among urban children whose mothers are illiterate and not working.

#### Prevalence of child undernutrition by interaction between paternal education and occupation

Table 6 demonstrates a cross-classification between education and paternal occupation, highlighting that children in rural and urban areas have the highest rates of stunting (43.1% vs. 47.5%), underweight (41.5% vs. 37.2%) and any form of undernutrition (20.0% vs. 14.1%) when their fathers are illiterate and engaged in agricultural or other professions. In addition, the prevalence of wasting reaches its peak among rural children whose fathers have similar standards, with a rate of 52.7%. On the other hand, the highest incidence of wasting is observed in urban children whose fathers are service owners or businessmen with no formal education (11.5%). Children in rural areas whose fathers have similar standards suffer the most from all form of nutrition (5.6%). Among both rural and urban children, whose fathers have secondary or higher education and are employed or run their own business, the incidence of undernutrition is extremely low, according to various nutritional assessments.

#### Probit regression coefficients of child nutrition by parental characteristics

To investigate the cumulative impact of paternal characteristics on various undernutrition indices, a binary probit regression model is used, as all undernutrition indices are binary responses. Probit analysis provides statistically significant results regarding the effects of parental characteristics on the likelihood of a child suffering from undernutrition. For this analysis, only those covariates found to be significant in the bivariate analysis are taken into account. Three separate models are applied in this case, namely Model 1, Model 2 and Model 3. Model 1 is not adjusted because it examined parental characteristics one by one. Model 2 and Model 3 are adjusted. While both models initially considered parental characteristics jointly, Model 3 included additional important covariates as potential confounding factors. The probit coefficients are presented in Table 7. The binary probit model has been shown to be effective in elucidating the prevalence of stunting in all three models, as well as the prevalence of underweight, wasting and any form of undernutrition in Model 1 and Model 2. All probit coefficients, with few exceptions, are negative in all three models. This means that the risk of stunting, wasting, underweight and any form of undernutrition decreases with positive changes in parental characteristics.

**Table 7:** Binary probit coefficient of paternal characteristics affecting nutritional status of children under five in urban and rural areas, BDHS 2017–18.

Type of undernutrition	Parental Characteristics	Model-1		Model-2		Model-3	
		Urban	Rural	Urban	Rural	Urban	Rural
Stunting	Mother’s education	-0.253^a^	-0.287^a^	-0.111^c^	-0.174^a^	-0.085^b^	-0.100^b^
	Father’s education	-0.298^a^	-0.268^a^	-0.208^a^	-0.184^a^	-0.174	-0.124^a^
	Mother’s working status	-0.201^b^	-0.115^b^	-0.104	-0.058^d^	-0.098	-0.007
	Father’s occupation	-0.349	-0.226^a^	-0.245^a^	-0.128^b^	-0.159^c^	-0.081^d^
Underweight	Mother’s education	-0.284^a^	-0.268^a^	-0.186^b^	-0.195^a^	-0.139^c^	-0.133^a^
	Father’s education	-0.251^a^	-0.203^a^	-0.138^b^	-0.111^a^	-0.088	-0.052^d^
	Mother’s working status	-0.240^b^	-0.064^d^	-0.161^c^	-0.016	-0.108	0.060
	Father’s occupation	-0.248^a^	-0.223^a^	-0.143^d^	-0.147^b^	-0.046	-0.112^c^
Wasting	Mother’s education	-0.191^b^	-0.063^d^	-0.171^b^	-0.044	-0.139^c^	-0.042
	Father’s occupation	-0.207^c^	-0.247^a^	-0.165^d^	-0.238^a^	-0.099	-0.226^a^
Any form of undernutrition	Mother’s education	-0.302^a^	-0.263^a^	-0.192^a^	-0.156^a^	-0.104^c^	-0.087^b^
	Father’s education	-0.269^a^	-0.244^a^	-0.146^b^	-0.162^a^	-0.109	-0.105^a^
	Mother’s working status	-0.219^a^	-0.071^c^	-0.132^c^	-0.016	-0.134^d^	0.038
	Father’s occupation	-0.327^a^	-0.286^a^	-0.223^b^	-0.203^a^	-0.104^c^	-0.164^a^

***Note*.** ^a^p<0.001. ^b^p<0.01. ^c^p<0.05. ^d^p<0.10. For all cases: Model-1, also called crude model, included the parental characteristics (education and occupation) one by one. For stunting, underweight and any form of undernutrition: Model-2 included parental education and occupation. Model-3 also added mother’s age, region, access to mass media, wealth index, mother’s age at first marriage, age at first birth, body mass index, child’s age, birth order, number of living children and birth interval as confounders along with parental characteristics. In running Model 3, the option to include missing values in SPSS was chosen because some values were missing in maternal BMI and birth interval. For wasting: Model-2 included mother’s education and father’s occupation only and Model-3 added wealth index, age at first birth and body mass index.

In Model 1 and Model 2, the probit coefficients for maternal and paternal education in both rural and urban areas are statistically significant. In Model 3, paternal education is insignificant in urban areas. The probit coefficients for underweight and any form of undernutrition are higher for mother’s education than for father’s education. For instance, when examining underweight in Model 1, the coefficients for father’s education are -0.251 in urban areas and -0.203 in rural areas, while those for mother’s education are -0.284 and -0.268, respectively. In the case of stunting, an opposite order is observed.

With few exceptions, the probit coefficients for maternal working status show consistent negativity in all three models. As an example, in Model 3, the coefficient for mother’s working status associated with any type of undernutrition in urban regions is -0.134. This suggests that children with working mothers are more likely to suffer from any form of undernutrition than those with non-working mothers. Unlike mother’s working status, the probit coefficients for father’s occupation are consistently negative across all three models. For example, children of fathers who are service holders or businessmen have a lower risk of suffering from stunting, underweight, wasting and any form of undernutrition than children whose fathers are farmer or other professionals.

## Discussion

In recent decades, Bangladesh has made significant progress in reducing child undernutrition through various intervention programs organized by the government and development partners. However, the present findings indicate that the prevalence of undernutrition among children under five remains high. Chowdhury et al. [31] reported that Bangladesh, Nepal and Pakistan have not achieved significant success in reducing severe child undernutrition compared to other South Asian countries. The limited success of this endeavour may be attributed to the lack of coordination among key sectors, such as government institutions, academic institutions, research and training organizations, and national and international NGOs [32]. It is important to ensure that these sectors work together effectively to achieve the desired outcome.

Child undernutrition is more prevalent in rural areas than in urban areas. Rahman and Rahman [27] came to similar conclusions when analysing data from the 2014 BDHS. In addition to differences between urban and rural areas, there are other factors associated with undernutrition that are shaped by the complex interactions between individuals and their social, cultural, economic, and environmental contexts [33]. The social determinants of health provide a conceptual framework for understanding the variations in health risks and outcomes within and between different population groups, including those residing in rural and urban areas [34]. This study therefore examined how parental characteristics influence the prevalence of undernutrition among children under five in rural and urban areas. It shows that paternal characteristics play a significant role in reducing child undernutrition in both rural and urban settings, with a particularly strong impact in urban areas. These findings are consistent with previous research conducted in Bangladesh [24] and Nigeria [11].

Children from both rural and urban areas whose mothers have secondary or higher education are less likely to suffer from stunting, underweight, wasting or any other form of malnutrition compared to children whose mothers have less or no formal education. Maternal education has a strong impact on stunting in rural areas and for other indices in urban areas. This difference persisted even after adjusting for factors such as maternal age and working status, paternal education and occupation, wealth index, access to mass media, religion, regional division, age at first marriage, age at first birth, body mass index, birth order, age of the child and birth interval. These findings are consistent with the results of several previous studies conducted in Bangladesh [19, 20, 35, 36]. Women with limited or no formal education face a higher risk of experiencing undernutrition [37], which can result in low birth weight babies and undernourished children [18]. In contrast, mothers with higher levels of education are more likely to follow prenatal and postnatal care and to prioritize the health and nutritional status of their children [38]. This is due to their extensive knowledge of child care.

Similar to maternal education, paternal education is negatively correlated with child stunting, underweight and any form of undernutrition, although it is not significantly associated with wasting. Hossain and Khan [21] reported similar findings in their investigation of child undernutrition in Bangladesh. The current study also found that the educational level of fathers plays a more important role than that of mothers in reducing the risk of undernutrition in children under five. In Bangladesh, women are traditionally responsible for household work and childcare, with limited in family decision-making. According to Naved [39], men with higher levels of education tend to have higher earning potential and often marry women with similar educational qualifications. Children whose parents have the same level of education are at the lowest risk of suffering from stunting, underweight and any form of undernutrition, regardless of whether they live in rural or urban areas. This may be due to the fact that couples with similar educational backgrounds tend to share financial responsibilities, household chores and childcare equally.

Previous studies have identified an association between maternal occupation and child malnutrition, including conditions such as stunting, underweight and wasting [26, 40]. However, this study’s findings only partially align with prior research. Specifically, maternal employment status is only significant in relation to child wasting in rural areas. Children of working mothers have a higher risk of suffering from wasting than children of non-working mothers. The phenomenon can be attributed to the prevalence of economic hardship, low levels of education and illiteracy among working women in rural areas. These factors often lead them to unskilled jobs, such as agricultural tasks and household chores. Additionally, they may lack the necessary knowledge on how to properly care for and raise children.

When considering paternal occupation as a single factor, children in both urban and rural areas whose fathers are in service or business have a lower risk of being stunted, underweight, or suffering from any form of undernutrition compared to children whose fathers work in agriculture or other professions. This trend persists in urban areas even after controlling for other background characteristics. Similar to this study, Utamia et al. [41] also observed an inverse relationship between the father’s employment status and the prevalence of stunting. Das and Gulshan [26] found that in Bangladesh, those employed in the service or business sector are generally wealthy and often well-educated. This enables them to efficiently monitor their family’s needs, ensure food security, maintain a healthy environment, and provide nutritious meals for all family members, especially children.

In urban areas, children whose fathers are employed or run their own business while their mothers take care of household chores are less likely to experience stunted growth, being underweight, or suffering from any form of undernutrition. Conversely, in urban areas, children with working mothers and fathers in service or business professions are at a higher risk of being underweight. This could be attributed to the challenge that working mothers face in finding sufficient time to care for children through a balanced diet. In addition, children with low-wage parents are the most susceptible to stunting and any form of undernutrition. One possible explanation is that most low-wage workers often come from disadvantaged backgrounds and have less or no formal education.

### Strengths and limitations

One strength of this study is its use of the nationally representative BDHS dataset, which allows the findings to be applicable to all children in both rural and urban areas of Bangladesh. Another notable strength is the consideration of various combinations of parental characteristics, including education and occupation for both mothers and fathers, in analyzing their influence on child undernutrition. As a result, the findings are quite interesting and unique. This study also has some limitations. Due to the use of a secondary dataset, this study has no control over the quality of data, measurement indicators, and variables selection. For example, BDHS does not include data on the diet, physical exercise, and smoking behaviour of parents, which were not analysed as potential confounders. Additionally, rapid urbanization may have caused errors in urban-rural calculations obtained from recent BDHS data compared to previous data.

## Conclusion

This study analysed data from BDHS 2017–18 to investigate the impact of parental characteristics on undernutrition prevalence among children under five in urban and rural areas of Bangladesh, while controlling for other potential risk factors. The findings indicate a significant number of undernourished children in Bangladesh, with a higher prevalence of undernutrition in rural areas compared to urban areas. The nutritional well-being of children is significantly influenced by their parents’ level of education and employment status. Therefore, it is crucial to provide education up to at least secondary level to all individuals in every household, regardless of their location, whether rural or urban. In fact, higher education improves an individual’s employment status and economic condition, which in turn leads to a reduction in child undernutrition.
